# Supplementary material for: CANDIDATE: A tool for generating anonymous participant-linking IDs in multi-session studies
Source: PLoS One. 2021 Dec 15;16(12):e0260569. doi: 10.1371/journal.pone.0260569 (PMC8673636; doi:10.1371/journal.pone.0260569)
Supplement: Attachment — Submitted filename: Review.pdf [file pone.0260569.s002.pdf]

1. I tried the code from GitHub. Using the htm file. There seems to be bug when entering the same input multiple times. I can at least produce 2 different id's. Further, If I add Symbols, Numbers letter to an existing string of solely latin-letters I receive the same ID, Only by adding another latin-character the ID changes. Although, this is no evaluation criteria for the paper it makes me wonder if these are just bugs or conceptual errors?
2. Non-latin letters are not treated as letters. A combination of Non-latin letters and latin letters leads to an reset of the application. I tried with Arabic letters.
3. Further from an UX POV I wouldn't let the user chose the ration as it is an error source. Better automatically set it to a reasonable value based on the max participants input.
4. "The privacy and safety of participants is of utmost importance in research that involves people. Privacy is also regulated by legislation such as the General Data Protection Regulations (GDPR) which applies in the European Union." → For research the more important and global codex for experiments is the Declaration of Helsinki. Pls refer to it.
5. Pls explain what a salt is on its first occurrence in the introduction.
6. The first 2 paragraphs in related work are motivational and should be integrated in the introduction.
7. "Experimentation showed that a suitable compromise between integrity and anonymity is achieved when N is ten times the anticipated number of participants" →This is anticipation of the Evaluation results. Pls consider removing or explicitly referring to the evaluation section.
8. It is misleading, that the author mostly speaks of the name as an input variable, although any String could be used. When read superficially one could be mislead that the tool is not suitable for larger studies where it is likely to have multiple participant with the same name. I would strongly advise to scatter the information that any unique string generated from easy accessible information can be used as an input. That's said: Pls add this as possible limitation, as it is up to the experimenters ensure such unique input strings.
9. Line 191 'L' and 'ID-List' are not previously defined
10. 191 hash-typefree = Find-free-slot(name, L, ID-List) → Isn't this bound to often return a value >9? Then switch in hash function hardly make any sense. This leads me to the point that I do not understand why there are 10 different digest generating function in the hash function.
11. I think Add-Function (Line 186) could be simplified by inverting the check in the if-statement.

```
186 Add(name, N)
187     ID_original = Encode(name, N, hash-type_default)
188     IF ID_original in ID-list
189         hash-type_free = Find-free-slot(name, L, ID-List)
192         ID_original = Encode(name, N, hash-type_free)
193         validation-code = Encode(name, N, hash-type_free + hash-type_offset)
194         ATTACH (hash-type_free, validation-code) TO ID_original


195     Add ID_original to ID-list
```

12. Line 194 souldn't it be $ID_{alternative}$
13. In add and lookup function '=' is used the other function use ':=' pls unify
14. "199 FOR EACH (hash-type, validation-code) ATTACHED TO IDoriginal" parameters in FOR EACH not previously defined
15. It is bad programming habit to have more than one return statement. Pls revise the code of Lookup function. I find it further strange, that this function returns a newly generated code in

case the FOR EACH runs without running into the THAN condition of the IF statement. Pls explain.

16. I understand why the Sanitize method removes these characters, however maybe an Experimenter relies on these removed characters in order to create unique input strings. In the input text field I would check for the removed characters and make them invalid input.
17. Code of Soundex function is missing
18. "245 (without the four-character length restrictions)" →Out of nowhere statement. What is this 4 char restriction?
19. Pseudo code of CONFIDENCE procedure is missing
20. References to the hash coding algorithms are missing (CRC32, etc.)
21. "The CANDIDATE anonymisation tool" section needs more structuring. Pls add meaningful subheading
22. General Comment: Pls add flow charts or sufficient UML diagrams for all algorithms described in section "The CANDIDATE anonymisation tool". It would allow for drastically shorten text, less repetition and far easier understanding.
23. A general comment: If the input string can be anything, then functions like Sanitize and Soundex are a bit meaningless, as they are intended to deal with names. They also seem to only work on latin-letter input. Also in the further description of CANDIDATE everything seems to be directed on handling names as input strings, although they are not unique and would be an unfavorable choice to take.
24. Line 274-290: In the pseudo code of the add function there is no loop. So I am not comprehending where "we search through an array of hash functions" should occur?
25. 296-311 are mostly providing information already given.
26. General Comment: Pls add the name list used to increase replicability. Should be as a supplement.
27. Table 4/5/6/7. Pls type out all numbers
28. Table 8: why is 'unused' column not present for N=10,000 and in Table7?
29. Table7/8: How can you explain, that min/mean values are the same in all conditions except for N=100?
30. General Question regarding Evaluation: From what I understood from the explanation of the CANDIDATE algorithm, Anonymity and Encoding success greatly depends on the truncated hash value. In this case, this parameter is not given in the Evaluation section, and it is not varied to evaluate its influence.
31. "With 10,000 slots (four-digit IDs) the smallest number of items per ID was 1, which indicates no anonymity. However, the mean number of hits per ID is 10.34 which is above the limit of acceptable anonymity" → This is a bit of an easy argumentation. In course of manuscript it is stressed multiple times, how important anonymity is and here the author is implying, that it is ok to not reach absolute anonymity.
32. Further, after reading the Evaluation section, for me there seems to be an inherent conflict between 'encoding success' and 'anonymity'. This is greatly impacting usability, as a user I shouldn't have to know/understand the details of the CANDIDATE algorithm in order to be able to choose the just correct coding space.
33. The biggest weakness IMHO is not mentioned: The phonebook attacking scenario implies that the attacker got hold of the anonymous data and tries to identify the individuals. However, if an attacker was able to obtain the research data why shouldn't s/he also be able to get hold of the study organization data where the participant's name are listed? So the whole method relies on the security of the names list (i.e. the input data). The link-table approach, of course, harbors the same problem of keeping the link-table securer. However,

even if an attacker should be able to obtain the participants name list and the anonymous data s/he could not link them.

34. A further limitation is that the whole evaluation was only conducted with names in Latin letters, whilst in Lines 166-175 it is explicitly stated that any kind of input string could be used. Unfortunately, therefore one can only consider the CANDIDATE tool validated for names provided in Latin-alphabet, of course considering the here found boundaries/limitations.
35. A further practical problem, though not a limitation of CANDIDATE itself, is, that the experimenter must pay great attention for possible errorness Input strings when generating the IDs.
36. “Evaluations show that the tool successfully assigns unique and anonymous IDs to participants” → Being picky, this is not true, as assigning unique and anonymous IDs depends on the correct set of parameters (as you write in following sentence).
