# Supplementary material for: CANDIDATE: A tool for generating anonymous participant-linking IDs in multi-session studies
Source: PLoS One. 2021 Dec 15;16(12):e0260569. doi: 10.1371/journal.pone.0260569 (PMC8673636; doi:10.1371/journal.pone.0260569)
Supplement: Attachment — Submitted filename: Review.docx [file pone.0260569.s004.docx]

1. Line 241: ≥ 9: digest := djb2(name + salt[8 - hash-type]) 🡪 Isn’t this bound to run in a run time error as hash-type will be at least 9 resulting in a negative index? However even if inverted “hash-type – 8” It could run into a runtime error as the value of hash-type is not constrained but the salt array has a pre-defined length. I know I am being picky here but as this is basically the core function of your whole approach the possibilities of runtime errors should be completely omitted. Therefore, a predefined salt-array with a fixed set of entries cannot be the solution. I would suggest to generate the salt generically during runtime.
2. REVIEWER #1 (pdf): 14. “199 FOR EACH (hash-type, validation-code) ATTACHED TO IDoriginal” parameters in FOR EACH not previously defined
   [RESPONSE]: The hash-type and validation-code in the FOR EACH are declared in the lines above (in the same IF block)
   🡪 As far as I see it the If statement where hash-type and validation-code are defined belong to the Add(…) function. The FOR EACH is part of the Lookup(…) so the variables defined in Add(…) should be unknown in Lookup(…).
3. REVIEWER #1 (pdf): 16. I understand why the Sanitize method removes these characters, however maybe an Experimenter relies on these removed characters in order to create unique input strings. In the input text field I would check for the removed characters and make them invalid input.
   [RESPONSE]: This is an excellent suggestion. An input check was added to the tool that now only allows variations on the Latin alphabet, numbers (in case of phone numbers), @ and dot (in case of e-mails), and hyphen (as used in some connected names). The manuscript was also updated to reflect this change.
   🡪 Unfortunately I was unable to detect this change is the manuscript. Could you please give me direction?
4. When looking at the hash function I was wondering if there is a reason why crc32 was not used for the letter shifted input names but only djb2?
5. I appreciate the added Fig.1 and Fig2. However, I think Fig2 is currently not really self-containing and is not really understandable. I think it would be good to also use concrete example as in Fig1 for explaining it.
6. REVIEWER #1 (pdf): 22. General Comment: Pls add flow charts or sufficient UML diagrams for all algorithms described in section “The CANDIDATE anonymisation tool“. It would allow for drastically shorten text, less repetition and far easier understanding.
   [RESPONSE]: A diagram illustrating the essence of the algorithm, namely the collision handling using multiple hash functions was added to the revised manuscript (as this is the core of the approach). A diagram illustrating the generation of the different hash functions was also added. An attempt was made to make it understandable for a wider readership as knowledge and experience is required to read and interpret UML diagrams.
   🡪 Here I disagree. One who can read AND understand the given pseudocode is highly likely able to comprehend flow charts or sufficient UML diagrams. Further, it will be a big help for colleagues who would like to build on your work. It took me properly 5-time to really think through the algorithm with just the pseudocode and the textual description then it would have been with proper diagrams. By the same time I am also less confident to not have missed a glitch or an error.
