# Supplementary material for: CANDIDATE: A tool for generating anonymous participant-linking IDs in multi-session studies
Source: PLoS One. 2021 Dec 15;16(12):e0260569. doi: 10.1371/journal.pone.0260569 (PMC8673636; doi:10.1371/journal.pone.0260569)
Supplement: Attachment — Submitted filename: response memo R3.docx [file pone.0260569.s005.docx]

# Response to reviewers’ comments on revision 2

## Reviewer #1

**[Reviewer #1]:**

1. Line 241: ≥ 9: digest := djb2(name + salt[8 - hash-type]) 🡪 Isn’t this bound to run in a run time error as hash-type will be at least 9 resulting in a negative index? However even if inverted “hash-type – 8” It could run into a runtime error as the value of hash-type is not constrained but the salt array has a pre-defined length. I know I am being picky here but as this is basically the core function of your whole approach the possibilities of runtime errors should be completely omitted. Therefore, a predefined salt-array with a fixed set of entries cannot be the solution. I would suggest to generate the salt generically during runtime.

**[RESPONSE]: The reviewer is right that there is a risk of a run-time error. The simulations shows that this is highly unlikely (see Table 2). With N = 1000 the hash-type value is never larger than 5, and with larger N’s it is lower. To generate a salt generically during run-time as suggested by the reviewer may also not be a feasible solution. From a theoretical perspective one may not be able to deterministically find a salt that results in a free slot that at the same time provides sufficient obfuscation. In any case the algorithm will detect if such a situation occurs and report to the experimenter that it was unable to encode a given participant. To make this situation more transparent to the reader I have added this as an exception in the pseudo-code, and also added a comment regarding this in the text (just before Table 2).**

2. REVIEWER #1 (pdf): 14. “199 FOR EACH (hash-type, validation-code) ATTACHED TO IDoriginal” parameters in FOR EACH not previously defined
   [RESPONSE]: The hash-type and validation-code in the FOR EACH are declared in the lines above (in the same IF block)
   🡪 As far as I see it the If statement where hash-type and validation-code are defined belong to the Add(…) function. The FOR EACH is part of the Lookup(…) so the variables defined in Add(…) should be unknown in Lookup(…).

**[RESPONSE]: Actually, the variables defined above were intended as global/state variables to the algorithm. I have adjusted the pseudo code with labels so that it is clearer which are internal state variables to the algorithm, constants, and what are variable parameters.**

3. REVIEWER #1 (pdf): 16. I understand why the Sanitize method removes these characters, however maybe an Experimenter relies on these removed characters in order to create unique input strings. In the input text field I would check for the removed characters and make them invalid input.
   [RESPONSE]: This is an excellent suggestion. An input check was added to the tool that now only allows variations on the Latin alphabet, numbers (in case of phone numbers), @ and dot (in case of e-mails), and hyphen (as used in some connected names). The manuscript was also updated to reflect this change.
   🡪 Unfortunately I was unable to detect this change is the manuscript. Could you please give me direction?

**[RESPONSE]:** This is perhaps a misunderstanding on my part. The change was done to the browser implementation (updated on GitHub), not the manuscript. In the currently revised manuscriptI have removed sanitize from the algorithm and written that the algorithm assumes sanitized names, and that sanitized names can be ensured using input checks in the user interface.

4. When looking at the hash function I was wondering if there is a reason why crc32 was not used for the letter shifted input names but only djb2?

**[RESPONSE]:** No, there was no reason for this. Testing showed that there were enough hash functions as is. If one needs more hash functions, all the pre-processing cases that are applied to djb2 (shifts and adding the salt) can also be applied to CRC32 with the desired effect. I have added a comment about this in the text (just before Table 2).

5. I appreciate the added Fig.1 and Fig2. However, I think Fig2 is currently not really self-containing and is not really understandable. I think it would be good to also use concrete example as in Fig1 for explaining it.

**[RESPONSE]: I have replaced Fig 2 with a new illustration (Fig. 3) that shows the steps involved in the encoding (Fig. 3 a, b, c, d, e, f, g and h). This figure builds on the example in Table 3 which now is obsolete. I have therefore removed Table 3 and 4.**

6. REVIEWER #1 (pdf): 22. General Comment: Pls add flow charts or sufficient UML diagrams for all algorithms described in section “The CANDIDATE anonymisation tool“. It would allow for drastically shorten text, less repetition and far easier understanding.
   [RESPONSE]: A diagram illustrating the essence of the algorithm, namely the collision handling using multiple hash functions was added to the revised manuscript (as this is the core of the approach). A diagram illustrating the generation of the different hash functions was also added. An attempt was made to make it understandable for a wider readership as knowledge and experience is required to read and interpret UML diagrams.
   🡪 Here I disagree. One who can read AND understand the given pseudocode is highly likely able to comprehend flow charts or sufficient UML diagrams. Further, it will be a big help for colleagues who would like to build on your work. It took me properly 5-time to really think through the algorithm with just the pseudocode and the textual description then it would have been with proper diagrams. By the same time I am also less confident to not have missed a glitch or an error.

**[RESPONSE]: I have added flow charts (see new Fig. 1 (a, b c and d).**

## Reviewer #2

**[Reviewer #2]**: The manuscript is greatly improved. The graphs help make the data more accessible, and the later sections of the paper do a really good job of explaining the limitations and appropriate use-cases for the tool.

**[RESPONSE]**: Thank you very much for these encouraging comments.

## General changes

I have added a paragraph in the introduction where the term “anonymous” is briefly discussed and reflected upon which I think is relevant for this study.
