# Supplementary material for: CANDIDATE: A tool for generating anonymous participant-linking IDs in multi-session studies
Source: PLoS One. 2021 Dec 15;16(12):e0260569. doi: 10.1371/journal.pone.0260569 (PMC8673636; doi:10.1371/journal.pone.0260569)
Supplement: Attachment — Submitted filename: responsememoR4.docx [file pone.0260569.s006.docx]

# Response memo

EDIROR: The figures are needs to improve. The quality of figures maybe improved with proper image editor.

RESPONSE: The issues raised by the reviewers have been fixed (see below).

Reviewer #1: Thank you very much for the revision. Just one minor Editing issues: Fig 1a and 1c need minor polishing so that the text is not intersected by lines and all arrows are straight.

RESPONSE: Fig 1a and 1c have been adjusted to avoid collisions between text and lines. All arrows have been straightened.

Reviewer #2: The revised manuscript is relatively easy to follow, the explicit examples in Figures 1 and 3 are good and the added discussion of anonymity is helpful. I have only two minor comments. One cosmetic issue is that the flow chart would look better and be easier to follow if the text inside the different flow diagrams in Fig 1a and 1c was resized to make it more readable. Second, for the the online version of the algorithm, it would be helpful to add a hyperlink to the github page (especially if the code is expanded or modified in the future.

RESPONSE: The problems in Fig 1a and 1c were fixed by making the boxes larger so ensure a consistent text size.

A link to the github page is added in the algorithm caption.
